# MIDO GDM: an innovative artificial intelligence-based prediction model for the development of gestational diabetes in Mexican women

**DOI:** 10.1038/s41598-023-34126-7

**Published:** 2023-04-28

**Authors:** Héctor Gallardo-Rincón, María Jesús Ríos-Blancas, Janinne Ortega-Montiel, Alejandra Montoya, Luis Alberto Martinez-Juarez, Julieta Lomelín-Gascón, Rodrigo Saucedo-Martínez, Ricardo Mújica-Rosales, Victoria Galicia-Hernández, Linda Morales-Juárez, Lucía Marcela Illescas-Correa, Ixel Lorena Ruiz-Cabrera, Daniel Alberto Díaz-Martínez, Francisco Javier Magos-Vázquez, Edwin Oswaldo Vargas Ávila, Alejandro Efraín Benitez-Herrera, Diana Reyes-Gómez, María Concepción Carmona-Ramos, Laura Hernández-González, Oscar Romero-Islas, Enrique Reyes Muñoz, Roberto Tapia-Conyer

**Affiliations:** 1 University of Guadalajara, Health Sciences University Center, 44340 Guadalajara, Jalisco Mexico; 2 Carlos Slim Foundation, Lago Zurich 245, Presa Falcon Building (Floor 20), Col. Ampliacion Granada, 11529 Mexico City, Miguel Hidalgo Mexico; 3 National Institute of Public Health, Universidad 655, Santa María Ahuacatitlan, 62100 Cuernavaca, Mexico; 4 Maternal and Childhood Research Center (CIMIGEN), Tlahuac 1004, Iztapalapa, 09890 Mexico City, Mexico; 5 Ministry of Health of the State of Guanajuato, Tamazuca 4, 36000 Guanajuato, Gto Mexico; 6 Ministry of Health of the State of Hidalgo, Fraccionamiento Puerta de Hierro, Avenida de La Mineria 103, 42080 Pachuca, Hidalgo, Mexico; 7 Department of Endocrinology, National Institute of Perinatology, Montes Urales 800, Lomas de Chapultepec, Miguel Hidalgo, 11000 Mexico City, Mexico; 8 School of Medicine, National Autonomous University of Mexico, Universidad 3004, Coyoacan, 04510 Mexico City, Mexico

**Keywords:** Computational models, Predictive medicine, Gestational diabetes

## Abstract

Given the barriers to early detection of gestational diabetes mellitus (GDM), this study aimed to develop an artificial intelligence (AI)-based prediction model for GDM in pregnant Mexican women. Data were retrieved from 1709 pregnant women who participated in the multicenter prospective cohort study ‘*Cuido mi embarazo’*. A machine-learning-driven method was used to select the best predictive variables for GDM risk: age, family history of type 2 diabetes, previous diagnosis of hypertension, pregestational body mass index, gestational week, parity, birth weight of last child, and random capillary glucose. An artificial neural network approach was then used to build the model, which achieved a high level of accuracy (70.3%) and sensitivity (83.3%) for identifying women at high risk of developing GDM. This AI-based model will be applied throughout Mexico to improve the timing and quality of GDM interventions. Given the ease of obtaining the model variables, this model is expected to be clinically strategic, allowing prioritization of preventative treatment and promising a paradigm shift in prevention and primary healthcare during pregnancy. This AI model uses variables that are easily collected to identify pregnant women at risk of developing GDM with a high level of accuracy and precision.

## Introduction

Gestational diabetes mellitus (GDM), a common medical complication of pregnancy, is defined as “diabetes diagnosed in the second or third trimester of pregnancy that was not clearly overt diabetes prior to gestation^1^”. The estimated global prevalence of GDM in 2021 was between 4.3% and 38.1%^2^. The prevalence in North America and the Caribbean is reported to be 20.7%^3^. In Mexico, the prevalence of GDM is estimated to be between 10 and 12%, though insufficient data are available to determine the national prevalence^4,5^. Pregnant women with GDM have an increased risk of both short- and long-term maternal and perinatal complications^6^. Short-term complications include perinatal death, preeclampsia, and neonatal respiratory distress syndrome, among others^6,7^. Long-term complications related to GDM for both mother and infant can include impaired glucose metabolism, type 2 diabetes, cardiovascular disease/hypertension, and obesity^6,8^. Currently identified risk factors for developing GDM include maternal overweightness and obesity, later age at childbearing, previous history of GDM, family history of type 2 diabetes, ethnicity, and high fasting glucose level at the first prenatal visit (> 5.6 mmol/L)^8,9^. Combinations of risk factors can also be predictive; for example, the combination of abdominal circumference > 91.5 cm, fasting glucose > 4.5 mmol/L, and a body mass index (BMI) > 38.6 kg/m^2^ greatly increases the risk of GDM^10^. Genetic risk factors for developing GDM are also being explored^11^.

Screening and diagnosis of GDM occurs between weeks 24 and 28 of pregnancy; the gold standard for GDM diagnosis is an oral glucose tolerance test (OGTT)^1^. Early detection and initiation of care for GDM are key to successful interventions. Initial treatment often involves non-pharmacological interventions such as dietary modifications and increased physical activity, followed by pharmacotherapy if initial efforts do not result in normoglycemia^8^. GDM treatment reduces serious perinatal morbidity and treatment of even mild GDM can reduce the risk of fetal overgrowth, shoulder dystocia, and cesarean delivery^12,13^. Intrauterine exposure to maternal diabetes is associated with an increased risk for type 2 diabetes and obesity^14–16^, although screening for GDM during pregnancy does not appear to improve childhood obesity risk^17,18^. Excessive growth of the fetal abdominal circumference, occurring between 20 and 28 weeks, precedes a GDM diagnosis, suggesting that the onset of fetal growth disorder occurs prior to the usual time of GDM screening (24 to 28 weeks)^19^. Given a high birth weight is associated with an increased risk of childhood obesity^20^, it is possible that some effects of GDM begin to manifest in the fetus prior to the recommended screening and that outcomes may improve with an earlier GDM diagnosis. A study comparing outcomes in high-risk women diagnosed with GDM early in their pregnancy (prior to 24 weeks) with those diagnosed from 24 weeks found a benefit for earlier GDM diagnosis. Earlier diagnoses resulted in a significantly lower new-born composite outcome frequency (hypoglycemia, birth trauma, neonatal intensive care unit/special care nursery admission, stillbirth, neonatal death, respiratory distress, and phototherapy) compared with a later GDM diagnosis^21^.

While early diagnosis of GDM is key to improving outcomes, maintaining the gold standard of universal OGTT for pregnant women is not sustainable in low-resource countries, as they often lack the infrastructure to provide universal access to facilities that can perform OGTT^22^. Additionally, there are barriers to performing the OGTT, including the inconvenience of a multi-hour appointment with multiple blood draws, difficulty in tolerating the test protocol, and the lack of understanding of the disease by healthcare workers and pregnant women^23^. GDM diagnostic capacity was reduced during the COVID-19 pandemic as OGTT testing has been difficult; several countries modified their diagnostic approach to GDM to limit long appointments^24^. Modified diagnostic approaches, such as the utilization of faster, less accurate testing, may result in underdiagnosis of GDM^25^. This lost diagnostic capacity during the COVID-19 pandemic was not limited to GDM. A study conducted in Spain found an average decline in new diagnoses of 31.1% in 2020 compared with 2019^26^.

Artificial intelligence (AI) is being applied in healthcare to facilitate solutions to complex real-world problems in this current era of digital health. As such, it is important to evaluate potential new ways to utilize AI-based technologies that have the potential to change the delivery of primary healthcare and preventative medicine. Early risk stratification by prediction modelling based on AI is expected to facilitate early detection of GDM in at-risk populations that would benefit from early intervention. The ability to predict the risk of developing GDM during pregnancy, which increases the risk of complications during pregnancy, represents a useful companion to therapeutic decision-making and patient education. Models currently developed to predict GDM use clinical parameters and serum biomarkers that are not always accessible (e.g., OGTT in pregnant women is not readily available in Mexico)^27–30^. A tool for predicting the risk of developing GDM that requires less medical equipment and fewer laboratory tests is needed to facilitate early interventions for women with a high risk of developing GDM. This would be particularly useful in low- and middle-income countries, where a lack of resources and trained personnel may result in placing a lower priority on the diagnosis and care of women with GDM^31^. The remarkable performance of AI in predicting the risk for other diseases, such as coronary heart disease among patients with type 2 diabetes mellitus^32^, cardiovascular disease^33^, and diabetic retinopathy^34^, suggests it may hold promise in applications to predict GDM risk. We aimed to develop an AI-based prediction model for risk of developing GDM among pregnant women in Mexico.

## Results

### Screening population

The *Cuido Mi Embarazo* (CME) study collected data for 1709 pregnant women in Mexico between April 2019 and May 2021. For this work we used information from 860 women (430 with GDM, and 430 without GDM). The sociodemographic and clinical characteristics according to GDM diagnosis are shown in Table 1. There were significant differences in the non-GDM and GDM groups for age, pregestational BMI, parity, family history of diabetes, BMI at study enrollment, random capillary glucose at study enrollment, and fasting plasma glucose measured between the 24th and 28th week of pregnancy (first OGTT measurement).

**Table 1:** Sociodemographic and clinical characteristics of the included pregnant women according to GDM status.

Characteristics	Non-GDM group (n = 430)	GDM group (n = 430)	*P* value
*Age categories (n %)*			
min – 20 years	59 (13.7)	31 (7.2)	0.0001
20.1 – 25 years	122 (28.4)	87 (20.2)	
25.1 – 34.9 years	203 (47.2)	223 (51.9)	
> 35 years	46 (10.7)	89 (20.7)	
*Pregestational BMI (n %)*			
min – 25	237 (55.1)	128 (30.0)	0.0001
25.1 – 30	130 (30.2)	184 (42.6)	
30.1 – 35	41 (9.5)	82 (19.0)	
> 35	13 (3.0)	36 (8.4)	
*Parity (n %)*			
Nulliparous	189 (44.4)	167 (38.6)	0.101
Multiparous	237 (55.6)	263 (61.4)	
Family history of diabetes mellitus (n %)	121 (28.1)	254 (58.8)	0.0001
Family history of hypertension	104 (24.2)	57 (13.3)	0.806
History of hypertension (n %)	1 (0.2)	1 (0.2)	0.637
Gestational week	19.5 ± 6.6	20.2 ± 6.8	0.211
*Enrollment BMI (n %)*			
min–25	160 (37.2)	105 (24.4)	0.0001
25.1–30	125 (29.0)	159 (36.7)	
30.1–35	47 (11.0)	79 (18.4)	
> 35	10 (2.3)	37 (8.6)	
Data not available	88 (20.5)	51 (11.9)	
Capillary casual glucose at enrollment (mg/dL)	95.4 ± 19.5	99.3 ± 17.0	0.078
OGTT fasting serum glucose (mg/dL)	77.7 ± 7.8	93.9 ± 10.9	0.0001
OGTT 1-h serum glucose (mg/dL)	116.2 ± 29.7	165.2 ± 38.6	0.0001
OGTT 2-h serum glucose (mg/dL)	99.8 ± 21.0	137.7 ± 33.8	0.0001

*BMI* body mass index, *GDM* gestational diabetes mellitus, *GW* gestational week, *OGTT* oral glucose tolerance test, Continuous variables are expressed as mean ± standard deviation, Chi-square test was used to evaluate qualitative variables, student’s *t* test was used to evaluate continuous variables.

### AI predictive model

Our model was named the Medición Integrada para la Detección Oportuna (MIDO) AI model for predicting gestational diabetes (MIDO GDM). The training data subset included 860 pregnant women, the validation data subset included 86, and the test data subset included 86 (corresponding to 10% of the data subset, respectively). The training of this model was used as a pilot for the MIDO GDM, which is expected to continuously improve as additional information becomes available. Table 2 shows the sensitivity and specificity of the MIDO GDM model at different cut-off points. Analysis of the risk distribution showed that the optimal cut-off point to provide an ideal balance for sensitivity and specificity was ≥ 0.40, which provided a sensitivity of 85.12% and a specificity of 67.40% with a rate of correct classification of 71.85%. The artificial neuronal network (ANN) reached a precision of 84.4%, 77.9%, and 75.7% in the training, validation, and test data subsets, respectively. The model was then used to estimate the risk for all pregnant women included in the cohort (n = 1709), achieving a precision of 70.28% and a sensitivity of 83.26%.

**Table 2:** Sensitivity and specificity of the MIDO GDM model at different cut-off points.

Cut-off point	Sensitivity (%)	Specificity (%)	Correct classification (%)
≥ 0.06	99.77	5.08	28.91
≥ 0.23	94.88	36.28	51.02
≥ 0.40	85.12	67.40	71.85
≥ 0.53	80.70	76.47	77.53
≥ 0.81	46.05	93.51	81.57
≥ 0.90	38.14	98.44	83.27

*MIDO GDM* Medición Integrada para la Detección Oportuna (MIDO) artificial intelligence model for predicting gestational diabetes mellitus.

**Figure 1:**
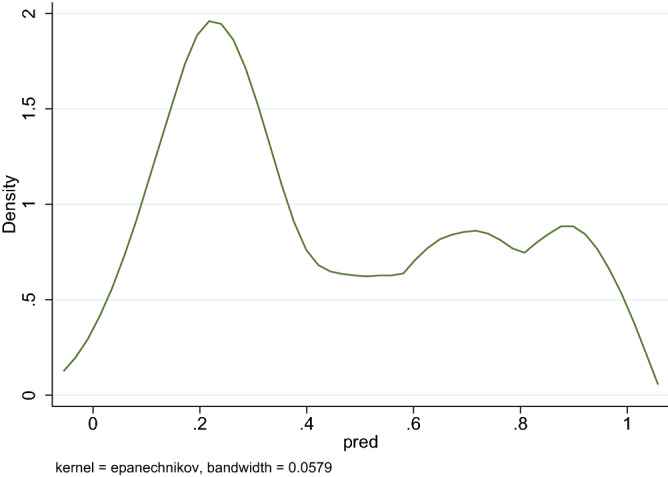
Kernel density plot.

**Figure 2:**
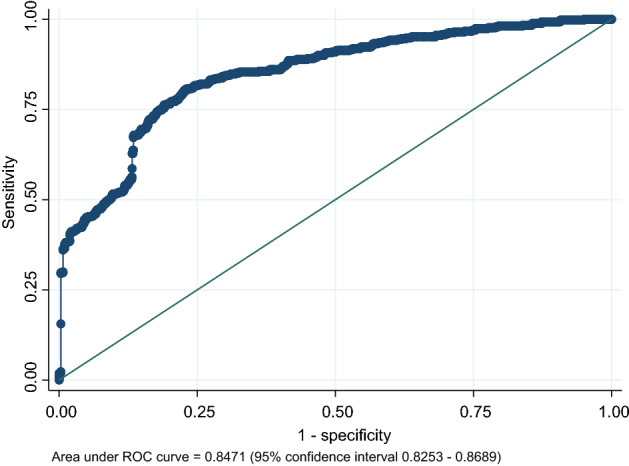
Area under the ROC curve for the MIDO GDM artificial neural network. *MIDO GDM* Medición Integrada para la Detección Oportuna (MIDO) artificial intelligence model for predicting gestational diabetes mellitus, *ROC* receiver operating characteristic.

A kernel density plot was generated using a bandwidth of 0.0579 to visualize the distribution of predicted values from the ANN model. The plot demonstrates a bimodal distribution, showing pregnant women with a lower risk of developing GDM concentrating on the left side of the plot and those with a higher risk concentrating on the right side (Fig. 1). The area under the receiver operating characteristic (ROC) curve on the test set was 0.8471 (95% confidence interval 0.8253–0.8689), indicating good accuracy of the model (Fig. 2).

External validation of the model showed an area under the ROC curve of 0.9308 with a precision of 86.00%, a specificity of 86.96%, and a sensitivity of 85.19%.

## Discussion

The current era of digital health provides an opportunity to change traditional models of primary healthcare. We have developed a novel digital health solution, MIDO GDM, which represents the first predictive model of GDM for pregnant Mexican women that is accurate in the first trimester of pregnancy, providing an opportunity for early detection that can be utilized prior to the use of the OGTT at 24 weeks. This approach provides an opportunity to offer personalized strategies to prevent the development of diabetes in pregnancy. Using MIDO GDM, pregnant women at risk of developing GDM can be identified in the first trimester of pregnancy with a group of easy-to-obtain variables. Thus, it would be possible to establish the risk of developing GDM in the early stages of pregnancy and to promote personalized preventive actions and appropriate treatment in a timely manner.

In our study, we identified nine variables that enabled the model to determine risk for developing GDM with a high level of precision, thus allowing us to propose an ANN that is simple to use and grants healthcare providers the opportunity to prioritize which patients receive preventative advice and treatment over time. Studies of early interventions (prior to pregnancy week 24) in women who are not yet diagnosed with GDM have reported promising results^35^. Among Finnish women who were < 20 weeks pregnant and at a high risk of developing GDM, those who received counselling on diet, physical activity, and weight control, and those who met with a dietician, had a significantly lower incidence of GDM compared with women who did not receive these interventions (*p* = 0.044)^36^. Another study that enrolled women with a BMI > 25 kg/m^2^ at their first trimester found that those enrolled in a Therapeutic Lifestyle Changes program, which included diet and mild physical activity, had a significantly lower incidence of GDM compared with those who received no intervention (*p* = 0.009)^37^. These studies support a potential benefit to the early identification of pregnant women at high risk for developing GDM. We propose that the MIDO GDM model be used ubiquitously throughout Mexico to improve the timing and quality of interventions for the prevention of GDM, a disease that is poorly diagnosed in the population.

We expect that MIDO GDM will play an instrumental role in changing the way primary care and preventative medicine are delivered. As an ANN, MIDO GDM will continue to learn as it is utilized as a part of the digital health platforms currently in use in Mexico. The MIDO GDM is expected to improve healthcare guidance by physicians who use MIDO Integral and to provide risk assessments and personalized health and lifestyle recommendations for patients who access the Mi Salud Integral app.

The success of a predictive model lies in its ability to predict, learn, and generate improvements that provide real-world value. As such, MIDO GDM will be incorporated within the MIDO healthcare strategy^38,39^, which is a series of interconnected digital platforms that enable healthcare professionals to proactively prevent obesity, type 2 diabetes mellitus, hypertension, and dyslipidemia at the community level (where laboratory tests are available)^39^. This ecosystem is known as MIDO Integral, and aims to provide timely and quality healthcare that is personalized to each person’s life course. For pregnant women, the MIDO Pregnancy system focuses on providing screening for diabetes, hypertension, weight gain during pregnancy, and urinary tract infections.

**Figure 3:**
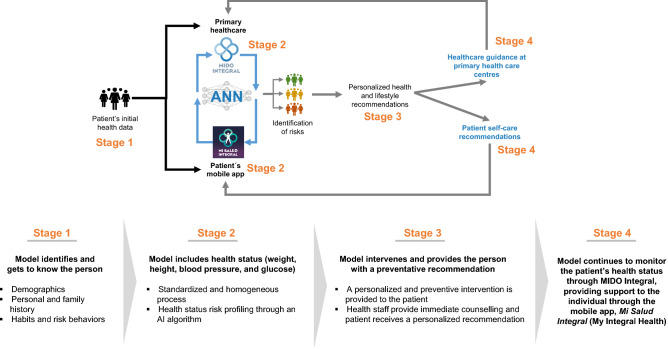
MIDO GDM as part of a proactive digital strategy for the detection of disease risks and disease. *AI*—artificial intelligence, *ANN*—artificial neuronal network, *MIDO GDM*—Medición Integrada para la Detección Oportuna (MIDO) artificial intelligence model for predicting gestational diabetes mellitus.

A diagram of the incorporation of the MIDO GDM model into the Mexican healthcare system is shown in Fig. 3. It is important that AI-based models do not merely remain conceptual but are integrated into healthcare systems. Because MIDO GDM is an ANN, the model will continuously learn as it is being used among the Mexican population within a functioning digital health ecosystem (MIDO Integral Ecosystem)^40,41^. Both healthcare professionals (via MIDO Integral) and patients (from home via the Mi Salud Integral app^42,43^) will have access to risk assessments and personalized health and lifestyle recommendations. This information will inform healthcare guidance and patient self-care.

MIDO Pregnancy is currently utilized across 388 primary healthcare centers in 32 states in Mexico, offering personalized recommendations to both healthcare professionals and patients based on their screening test results. Recommendations are made on the basis of decision trees and are given at the end of the assessment based on the results of the screening. Screening is performed at every visit to the health center and therefore our predictive model can be used to detect the risk of GDM from the very first prenatal visit in order to provide personalized GDM-prevention strategies as early as possible. MIDO GDM will also be able to offer and receive information from the mobile application "Mi Salud Integral". We expect that the application of this strategy will benefit primary healthcare and, in a larger scope, change public policy and shift the traditional primary healthcare models in Mexico and other countries.

The MIDO GDM model is simple and easy to implement even in low-resource settings, thus enhancing the practice of precision medicine. There is increasing evidence that digital health tools have the potential to improve patient health in a variety of clinical areas for those living in low- and middle-income countries, though it is widely recognized that the opportunities to use digital health tools are underutilized^40,44–46^. Under these settings, there is limited per capita spending on healthcare services, leaving many people without access to healthcare^40^.

This study had several strengths. First, we used variables with commonly and easily collected data, which made it feasible to collect sufficient data to develop this predictive model. The utilization of easily accessible data means that our model can be applied in low-resource settings, where frequent laboratory testing is not always feasible due to resource limitations. Second, we developed this model using data collected as part of a multicenter cohort study, in which all pregnant women received OGTT according to both national and international standards. Third, we used ANNs to develop our model, which are not only able to discover patterns and relationships in large amounts of data, they are also able to be trained to produce the desired input–output relationship by running the parameters to adjust the network behavior^47^. This gives them an advantage over conventional computing methods. Finally, our ANN model performs similarly or better than several models recently developed to provide early prediction of GDM in pregnant women. Our model had an AUC, sensitivity, and specificity of 0.8471, 83.26%, and 70.28%, respectively. A predictive model using a deep neuronal network reported respective values of 0.80, 63%, and 82%^46^. Similar values were also observed when applying a logistic regression model (AUC, 0.64–0.77; sensitivity, 58–69%; specificity, 53–86%)^48–50^ or a random forest model (AUC, 0.777; sensitivity, 65.1%; specificity, 81.3%)^49^.

This study also had several limitations. The model was based on a small set of data from Mexican women; therefore, the generalizability of the model to the entire population of Mexican women and other ethnic populations is unknown. Given the promising precision and sensitivity of the model, studies further validating this model using larger datasets are warranted. Aside from the limitations of the study, the model itself also has several limitations. First, some variables may be unavailable for some women; however, the effects of this may be minimized by imputation of the missing information. For example, although over 50% of women had missing values for glucose level at the time of their first pregnancy consultation, this variable is of great relevance for prediction. Unfortunately, in Mexico there are several barriers to routinely providing glucose testing at the first pregnancy consultation; thus, we were not surprised to see that data for this parameter were missing for many pregnant women. Considering these factors, we considered it appropriate to retain this variable by imputation of the missing information. Second, although false positives are likely, a positive readout will trigger advice on non-pharmaceutical preventative strategies that benefit all pregnant women rather than a pharmaceutical intervention. Third, tools such as MIDO GDM may not be easy for healthcare professionals to adopt. Finally, the usefulness of this tool will be dependent upon whether pregnant women successfully adopt preventative measures when advised.

In conclusion, we utilized ANNs to build a model that could predict the risk of developing GDM using variables for which data is commonly and easily collected. In building this model, we aimed to alleviate some of the uncertainty in the therapeutic decision-making process for pregnant women in Mexico and to assist physicians in making decisions related to clinical care. Multiple ANN algorithms were used to develop this model, which we expect can be used to identify at-risk pregnant women during the early stages of pregnancy, despite the limited availability of OGTT in low-resource settings. Early detection of GDM is critical for initiating timely preventative measures and successful interventions. Furthermore, it is hoped that this AI-based neural network will potentially play an instrumental role in changing the way primary care and preventative medicine is delivered.

## Methods

This study was conducted to build and train a neural network AI algorithm to predict the risk of developing GDM in Mexican women using socio-demographic and clinical variables, which were considered relevant for the detection of GDM risk. All women provided written informed consent prior to participation in the CME study. The study protocol was approved by the Research and Ethics Committees of the Secretary of Health of Hidalgo State (FSSA2018076) and the Secretary of Health of Guanajuato State (CONBIOETICA-11-CEI-003–20,190,704). The study was conducted in accordance with the Declaration of Helsinki, the International Conference on Harmonization guidelines for Good Clinical Practice, and applicable local laws and regulations. The study was registered at researchregistry.com (researchregistry7405).

### Data sources

The study population was derived from a prospective multicenter cohort study called CME [researchregistry7405]; this included data for 1709 pregnant women in Mexico, collected between April 2019 and May 2021. The CME study aimed to achieve detection of GDM in six primary healthcare centers in Mexico and to provide appropriate and timely care for GDM. Data were collected using standardized questionnaires for socio-demographic characteristics (address, type of medical affiliation, marital status), medical history (family and personal history of pathologies, blood type, gynecological history, medications used), regular medical visits (maternal weight, blood pressure, urine test strip [glucose/protein/pH/blood/ketones/bilirubin/nitrite/urobilinogen/specific gravity/leukocytes], alarm signs), laboratory data (hemoglobin, hematocrit, serum platelet level, serum glucose level, lipid profile, urine pregnancy test at each trimester), pregnancy resolution (date pregnancy ended, delivery method, number of newborns, complications during delivery), and management of self-monitoring for GDM (data related to at-home glucose monitoring using a capillary glucometer provided to participants of the cohort study). All participants in this cohort were referred for GDM testing. Clinical evaluation, including a 2-h 75-g OGTT that was performed between weeks 24 and 28 of their pregnancy in accordance with International Association of Diabetes and Pregnancy Study Group criteria^51^, was used to establish a GDM diagnosis, which was given if any single value met or exceeded normal glucose threshold values specified as normal for pregnant Mexican women (fasting value of 92 mg/dL [5.11 mmol/L]; a 1-h value of 180 mg/dL [10.0 mmol/L]; or a 2-h value of 153 mg/dL [8.49 mmol/L])^1,51^. In this analysis, we included pregnant women without type 2 diabetes and pregnant women who were at < 36 weeks gestation from the CME cohort. Furthermore, those diagnosed with pregestational diabetes, had multiple pregnancies, or had a previous chronic disease that required their pregnancy to be monitored by secondary care were excluded.

### Predictive model development

The use of AI, and ANNs specifically, has been investigated in a variety of healthcare applications, including clinical diagnosis, disease prediction and detection, risk stratification, and image analysis and interpretation^52–55^. More recently, ANNs are being used to inform healthcare management decisions with a goal of moving towards value-based care^55^. We used an ANN approach to develop our predictive model, which has advantages over traditional statistical methods, including the ability to work with non-structured and missing input data, the ability to identify complex non-linear relationships between predictive variables, parallel data processing, and the ability to learn^47,56^. A “weight” analysis can be used as a sensitivity analysis to explain relationships between input and output variables^57^. This type of analysis computes, quantitatively, the strength of connections between input and output factors. It is expected that this characteristic of the ANN approach will result in a high degree of predictive accuracy. Thus, ANN is ideal for developing a model to predict the risk of developing GDM.

We took the following steps to develop our ANN model: (i) data pre-processing, (ii) building and training the model, (iii) simplifying the model, adding dropout and weight regularization to address overfitting, and (iv) hyperparameter tuning. First, all candidate predictive variables that were selected after the systematic review were included to build a base model. Then, we conducted a machine-learning variable selection method to select variables that ensured better model discrimination to create an efficient approach for clinical practice with fewer redundant variables. We evaluated variables with predictive power that could be easily collected by questionnaire and did not require laboratory measurements. This strategy was employed to ensure the model could be used in situations where laboratory measurements were not readily available. We selected nine variables for GDM prediction after reviewing 28 published studies that reported findings from cohort and case–control studies, diagnosis or assessments of GDM, or development of predictive or risk models^27–30,58–81^ (listed and summarized in Table S1 and Figure S1) and in accordance with the information available for the CME study. The selected variables included maternal age (years); family history of type 2 diabetes (yes/no); previous diagnosis of hypertension (yes/no); BMI (kg/m^2^); gestational week at first prenatal visit; parity (number); birth weight of last child (grams); type of capillary glucose measurement at first prenatal visit (random/fasting); and capillary blood glucose level at first prenatal visit (mg/dL).

Missing data for each variable were imputed with the average, bordering, or null value observed in a typical pregnancy for a Mexican woman based on information provided by the Mexico Births Information Subsystem (SINAC) for the year 2020 (Table 3)^40^. All variables were standardized using the mean and standard deviation to be included into the model. The dataset for model building was first balanced to equalize the number of women with and without GDM, and then it was divided into subsets for training (80%), validation (10%), and testing (10%).

**Table 3:** Values used to impute missing data.

Input variable	Value^†^	Missing values (% of total)
Age, years	26	11.8
Family history of diabetes^‡^	0	0.2
Family history of hypertension^‡^	0.5	12.3
BMI before pregnancy, kg/m^2^	25	13.9
Gestational week at time of first pregnancy consultation	18	11.8
Number of gestations	1	0.5
Birth weight of the last child, g	3100	43.3
Type of glucose test^§^	0.5	64.1
Glucose level at time of first pregnancy consultation, mg/dL	92	61.5

*BMI* body mass index.

^†^Average value reported for a typical pregnancy in Mexico. ^‡^No = 0, yes = 1. ^§^Fasting = 1 or random = 0.

**Figure 4:**
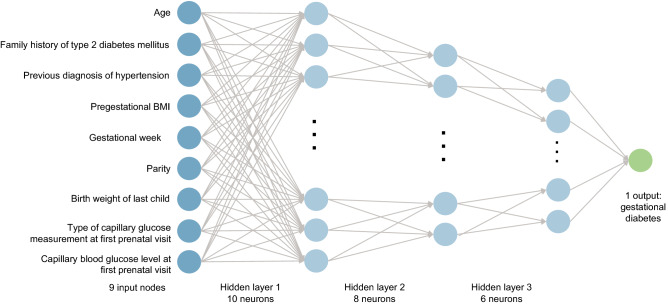
Artificial neural network architecture. *BMI*—body mass index, *GDM*—gestational diabetes mellitus.

Figure 4 shows the ANN architecture of the model. The model had a single output node, defined as the GDM diagnosis (yes/no), which was modelled using an ANN approach with the following hyper-parametrization: three hidden layers of 10, 8, and 6 nodes; Rectified Linear Activation as the hidden layer activation function; sigmoid or logistics as the output activation function; and a binary cross-entropy as loss of function to measure how far away from the true value the prediction was to allow for tuning of the parameters based on this value. Next, we used Adam, an adaptive learning rate optimization algorithm and set the number of epochs (i.e., the number of times the entire training dataset is shown to the network during training) as 100 and the batch size (i.e., the number of sub-samples given to the network after the parameter update occurs) as 50 using the Early Stopping technique. Finally, we assessed the accuracy of the model to predict GDM.

After obtaining the risk predictions, we performed an analysis of the risk distribution to define the optimal cut points that would provide an ideal balance for sensitivity and specificity. Analyses were performed using the Pandas, Numpy, and Keras (backend on tensorflow) modules in Python 3.6 (Python Software Foundation, Fredericksburg, VA, USA).

### External validation of the model

The model was externally validated using data collected during routine health care provided to pregnant women in Mexico City who visited the Health Units of the Maternal and Infant Research Center of the Birth Study Group (CIMIGEN [acronym in Spanish])^82^. Data were collected from the randomly selected records of 27 pregnant women with GDM and 23 pregnant controls without GDM who had their first medical appointment at ≤ 32 weeks of pregnancy. Data related to the variables used in the model were retrieved, including maternal age, family history of type 2 diabetes, previous diagnosis of hypertension, pregestational BMI, week of pregnancy at prenatal visit, parity, birth weight of last child, type of capillary glucose measurement at first prenatal visit (random/fasting), and capillary blood glucose level at prenatal visit. The selected pregnant women were confirmed to not be part of the cohort used to develop the predictive model. The resulting database (Supplementary Data S1) was carefully reviewed, and the completeness and plausibility of the dataset were confirmed prior to running the model.

## Supplementary Information

Supplementary Information 1.

Supplementary Information 2.

Supplementary Information 3.

## Data Availability

All data collected for this study will be made available upon reasonable request to the corresponding author.
