# Supplementary material for: MIDO GDM: an innovative artificial intelligence-based prediction model for the development of gestational diabetes in Mexican women
Source: Sci Rep. 2023 Apr 28;13:6992. doi: 10.1038/s41598-023-34126-7 (PMC10144896; doi:10.1038/s41598-023-34126-7)
Supplement: Supplementary file 1 — Supplementary Information 1. [file 41598_2023_34126_MOESM1_ESM.pptx]

## Slide 1
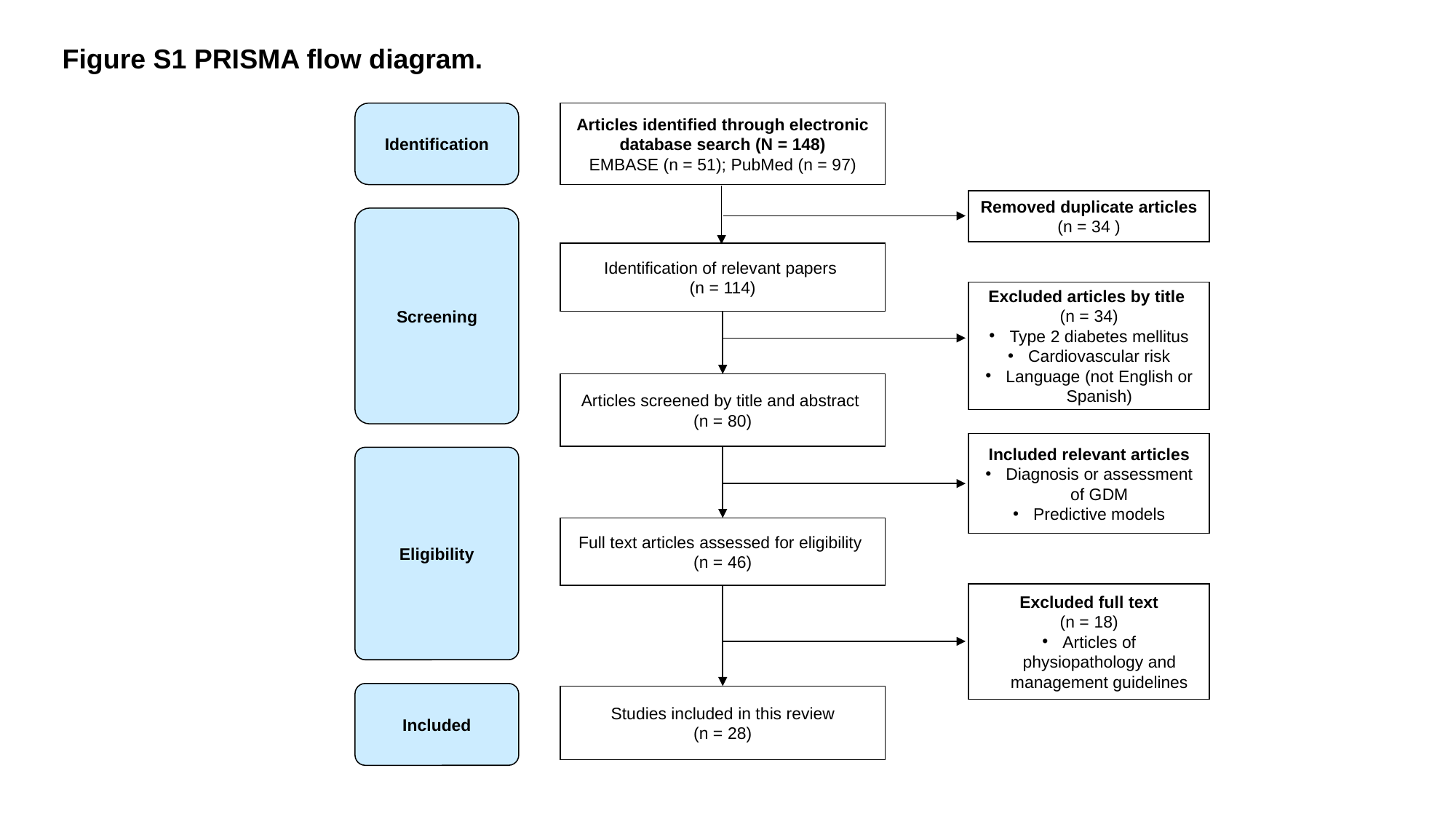

Figure S1 PRISMA flow diagram.
Identification
Articles identified through electronic database search (N = 148)
EMBASE (n = 51); PubMed (n = 97)
Removed duplicate articles
(n = 34 )
Screening
Identification of relevant papers
(n = 114)
Excluded articles by title
(n = 34)
Type 2 diabetes mellitus
Cardiovascular risk
Language (not English or Spanish)
Articles screened by title and abstract
(n = 80)
Included relevant articles
Diagnosis or assessment of GDM
Predictive models
Eligibility
Full text articles assessed for eligibility
(n = 46)
Excluded full text
(n = 18)
Articles of physiopathology and management guidelines
Included
Studies included in this review
(n = 28)
