# Supplementary material for: MIDO GDM: an innovative artificial intelligence-based prediction model for the development of gestational diabetes in Mexican women
Source: Sci Rep. 2023 Apr 28;13:6992. doi: 10.1038/s41598-023-34126-7 (PMC10144896; doi:10.1038/s41598-023-34126-7)
Supplement: Supplementary file 3 — Supplementary Information 3. [file 41598_2023_34126_MOESM3_ESM.docx]

**Table S1 Description of studies included in the literature review.**

| # | First author | Year | Country | Total No. Women | GDM Cases | | Risk predictors (categories) | |
| --- | --- | --- | --- | --- | --- | --- | --- | --- |
| 1 | Naylor, D^56^ | 1997 | Canada | 3131 | 113 | Maternal age (<30 y, 31–34 y, >35 y), BMI (<22.0, 22.1–25.0,> 25.1 kg/m^2^), race (White, Black, Asian, and other) | |  |
| 2 | Caliskan, E^57^ | 2004 | Turkey | 4612 | 143 | Maternal age >25 y, BMI >25 kg/m^2^, prior adverse obstetric outcome, family history of diabetes mellitus, prior macrosomic fetus | |  |
| 3 | van Leeuwen, M^27^ | 2010 | Netherlands | 995 | 24 | Maternal age (per year), BMI (per kg/m^2^), ethnicity non-Caucasian, family history of diabetes, smoking, previous miscarriage, history of GDM, parity, history of perinatal death  GDM = 1/[1 + exp(− β)], in which β is calculated as [− 6.1 + (0.83 × non-Caucasian ethnicity) + (0.57 × family history of diabetes mellitus) − (0.67 × multipara without history of GDM) + (0.5 × multipara with history of GDM) + (0.13 × BMI)] | |  |
| 4 | Nanda, S^26^ | 2011 | London | 11 464 | 297 | Maternal age (per year), BMI (per kg/m^2^), racial origin (Caucasian, African, south Asian, east Asian, and mixed), family history of diabetes, parity, cigarette smoking, conception method, adiponectin, sex hormone-binding globulin | |  |
| 5 | Teede, HJ^25^ | 2011 | Australia | 4276 | 356 | Maternal age (<25 y, 0 points; 25–34 y, 1 point; ≥35 y, 2 points), BMI (<29 kg/m^2^, 0 points; 30–34.9 kg/m^2^, 1 point; ≥35 kg/m^2^, 2 points), ethnicity (Anglo-Australian, European or other, 0 points; Polynesian, Maritime Southeast Asian, Chinese Asian, southern Asian or African, 1 point; mainland Southeast Asian, 2 points), family history of diabetes mellitus (1 point), history of GDM (2 points). A score of ≥4 was the suggested cut-off point | |  |
| 6 | Hedderson, MM^58^ | 2011 | USA | 580 | 199 | Mild hyperglycemia (<100, 100–140 mg/dL), hypercholesterolemia (<200, >200 mg/dL), blood pressure (normal, pre-hypertension), overweight/obese (BMI <25, >25 kg/m^2^) | |  |
| 7 | Göbl, CS^59^ | 2012 | Austria | 875 | 253 | History of GDM, history of prediabetes, previous recurrent abortions, glycosuria (>2.22 mmol/L), previous birthweight >4,500 g, age (per year), preconception overweight/obesity, relative with type 2 diabetes, previous preterm delivery <37 weeks, high-risk ethnicity, weight gain >10 kg during pregnancy, preconception hypertension, preconception dyslipidemia, fasting plasma glucose | |  |
| 8 | Trujillo, J^60^ | 2014 | Brazil | 4926 | 767 | Fasting plasma glucose (80, 85, 90, 92 mg/dL) | |  |
| 9 | Fong, A^61^ | 2014 | USA | 526 | 55 | HbA1c (<5.7%, 5.7%–6.4%) | |  |
| 10 | Harrison, CL^62^ | 2014 | Australia | 224 | 51 | Previous GDM, family history of type 2 diabetes mellitus, high risk ethnic group (south of east Africa, maritime southeast Asia, Polynesia, Chinese Asia, southern Asia, and southeast Asia), maternal age (<25 y, 25–34 y, >35 y), BMI (20–34.9, >35 kg/m^2^) | |  |
| 11 | Thériault, S^63^ | 2014 | Canada | 7929 | 381 | Maternal age (per year), BMI (per kg/m^2^), ethnicity, family history of GDM, history of GDM, macrosomic infant, adverse obstetric outcomes (GDM, gestational hypertension, recurrent spontaneous abortion, fetal death) | |  |
| 12 | Syngelaki, A^28^ | 2015 | United Kingdom | 73 334 | 1827 | Maternal age (per year), weight (kg), height (cm), racial origin (Caucasian, African, south Asian, east Asian, and mixed), family history of diabetes, use of ovulation drugs, birth weight, history of GDM | |  |
| 13 | Berggren, EK^64^ | 2015 | USA | 300 | 39 | Maternal BMI (kg/m^2^), first trimester serum hsCRP (mg/L) | |  |
| 14 | Capula, C^65^ | 2016 | Italy | 2654 | 555 | Constant − 2.2532 * (age/10) + 0.4128 * (age/10)2 + 0.0795 *pre-pregnancy BMI (kg/m^2^) | |  |
| 15 | Sweeting, AN^66^ | 2017 | Australia | 980 | 248 | Previous GDM, race (east Asian, south Asian), family history of diabetes, parity, maternal age (per year), BMI (per kg/m^2^) | |  |
| 16 | Bozkurt, L^67^ | 2018 | Austria | 216 | 82 | Leptin (ng/mL), adiponectin (mg/mL) | |  |
| 17 | Hinkle, SN^68^ | 2018 | USA | 2802 | 107 | Maternal age (per year), race-ethnicity, pre-pregnancy overweight or obesity, family history of diabetes, GDM in a prior pregnancy, nulliparity and HbAc1 (per 0.1%) | |  |
| 18 | Sweeting, AN^69^ | 2018 | Australia | 980 | 248 | Previous GDM, race (east Asian, south Asian), family history of diabetes, parity, maternal age (per year), BMI (per kg/m^2^), PAPP-A (logMoM), triglycerides (logMoM), lipocalin-2 (logMoM), interaction between triglyceride and lipocalin-2 (logMoM) | |  |
| 19 | Balani, J^70^ | 2018 | United Kingdom | 302 | 72 | Maternal age (per year), weight, BMI (kg/m^2^), percentage body fat, visceral fat mass, lean body mass, history of polycystic ovarian syndrome, family history of diabetes, history of hypertension, previous macrosomia | |  |
| 20 | Pezeshki, B^71^ | 2019 | Iran | 356 | 30 | HbA1c (5.35% at 24–28 gestational weeks, 5.75% at 20­–24 gestational weeks), BMI (>27), maternal age (>25 y), blood pressure (>140/90 mmHg) | |  |
| 21 | Zheng, T^72^ | 2019 | China | 4771 | 612 | Maternal age (>29 y), BMI (>27 kg/m^2^), fasting plasma glucose (>4.4 mM), triglycerides (>2.26 mM) | |  |
| 22 | Zhu, Y^73^ | 2019 | USA | 1750 | 115 | Maternal age >35 y, race/ethnicity (Asian/Pacific Islander, African American, Hispanic, other), pre-pregnancy overweight (BMI 23 to 25 kg/m^2^), previous GDM, pre-existing hypertension, waist-to-hip ratio (0.85), waist circumference (80 cm) | |  |
| 23 | Falcone, V^74^ | 2019 | Austria | 574 | 103 | Maternal age (per year), pregestational BMI (per kg/m^2^), fasting plasma glucose (mg/dL), fasting insulin (mU/mL), fasting C-peptide (ng/mL), HbA1c (mmol/mol), homeostatic model assessment of insulin resistance (dimensionless), homeostatic model assessment of beta-cell function, quantitative insulin sensitivity check index from insulin, quantitative insulin sensitivity check index from C-peptide, insulinogenic index from insulin, insulinogenic index from C-peptide | |  |
| 24 | Donovan, BM^75^ | 2019 | USA | 771 140 | 48 608 | Nulliparous, race/ethnicity (White, Hispanic, Black, Asian, Hawaiian/Pacific Islander, other), ﻿age at delivery (natural cubic spline transformed), pre-pregnancy BMI (natural cubic spline transformed), family history of diabetes, pre-existing hypertension | |  |
| 25 | Punnose, J^76^ | 2019 | India | 2275 | 578 | HbA1c (<5.2%, 5.2%–5.5%, >5.5%), previous GDM, family history of diabetes, gravidity (>1), mean corpuscular volume (per unit fL), hemoglobin (per mg/dL), BMI (per kg/m^2^), maternal age (per year) | |  |
| 26 | Xiong, Y^77^ | 2020 | China | 490 | 215 | Direct bilirubin (mg/dL) and fasting plasma glucose levels (mg/dL), prothrombin time, activated partial thromboplastin time | |  |
| 27 | Feng, P^78^ | 2020 | China | 13 448 | 2973 | Maternal age (>27 y), education (junior middle school or below, senior high school, college or higher), gravidity (>2), parity, baseline BMI (>24 kg/m^2^), gestational weight gain (>15 kg), systolic blood pressure (>110 mmHg), baseline FPG (mmol/L), WBC count (<7.7 WBC 10^9^/L), hemoglobin (g/L), eGFR (mL/min per 1.73 m^2^), BUN (<2.4, 2.4–2.9, 2.91–3.5, >3.5 mmol/L) | |  |
| 28 | Zhang,YZ^79^ | 2020 | China | 4421 | 414 | Maternal age (per year), pre-pregnancy BMI (kg/m^2^), family history of diabetes, polycystic ovary syndrome, history of GDM, systolic blood pressure, HbA1c, triglycerides, total cholesterol, LDL‑cholesterol, alanine transaminase, aspartate transaminase, gamma transglutaminase, visfatin, hsCRP, visceral fat thickness, subcutaneous fat thickness levels | |  |

*BMI* body mass index, *BUN* blood urea nitrogen, *eGFR* estimated glomerular filtration rate, *FPG* fasting plasma glucose, *GDM* gestational diabetes mellitus, *HbA1c* glycosylated hemoglobin, *hsCRP* hypersensitive C-reactive protein, *LDL* low-density lipoprotein, *MoM* multiple of the median, *PAPP-A* pregnancy-associated plasma protein, *WBC* white blood cell.
